# Inter simple sequence repeat markers to assess genetic diversity of the desert date (*Balanites aegyptiaca* Del.) for Sahelian ecosystem restoration

**DOI:** 10.1038/s41598-020-71835-9

**Published:** 2020-09-11

**Authors:** Selouka Mint Abdelaziz, Leila Medraoui, Mohammed Alami, Ouafae Pakhrou, Meryem Makkaoui, Ali Ould Mohamed Salem Boukhary, Abdelkarim Filali-Maltouf

**Affiliations:** 1 Laboratory of Microbiology and Molecular Biology, Faculty of Sciences, Mohammed V University, Rabat, Morocco; 2 Université de Nouakchott Al-Aasriya, Faculté des Sciences et Techniques, Laboratoire santé, environnement et société (LE2S), Unité de recherche génomes et milieu (GEMI), Nouakchott, Mauritanie

**Keywords:** Natural variation in plants, Plant molecular biology

## Abstract

Drought and desertification are the major environmental constraints facing the Sahelian agro-ecosystems for decades. Assessing genetic diversity of native tree species is critical to assist ecosystems restoration efforts. Here we describe genetic diversity and structure of seven *Balanites aegyptiaca L*. natural populations distributed across the Sahelian-Saharan zone of Mauritania using 16 polymorphic ISSR primers. These generated 505 polymorphic bands. Polymorphism information content (PIC) varied from (0.13–0.29) with an average 0.23, marker index (MI) averaged 7.3 (range 3.3–10.3) and resolving power (RP) ranged from (4.53–14.6) with an average 9.9. The number of observed alleles (Na) ranged from (0.62–1.39), Effective number of alleles (Ne) varied from (1.26–1.37), Shannon’s information index (I) ranged from (0.25–0.36). AMOVA analysis showed that 80% of the genetic variation was fined within populations, which is supported by a low level of genetic differentiation between population (GST = 0.21) and an overall estimate of gene flow among populations (Nm = 1.9). The dendrogram based on Jaccard's similarity coefficient and the structure analysis divided the seven populations into two main clusters in which two populations from the Saharan zone were grouped. Our results provide baseline data for genetic conservation programs of this Sahelian neglected crop and with an important econ-ecological role.

## Introduction

*Balanites aegyptiaca* Del. belongs to *Zygophyllaceae* family, commonly known as desert date tree, is a long-lived dicotyledonous spiny tree up to 10 m tall that is widely distributed in arid and semi-arid regions in Africa and South Asia^1–3^. In Mauritania, *B. aegyptiaca* is widespread throughout the country. It is a xerophytic tree distributed in Sahelian as well as the Saharan zones. The Desert date is a multipurpose tree offering protection against desertification, provides food for human and animals in addition to many medicinal uses^2,3^. For instance, fruit of *B. aegyptiaca* (locally known as *Tooga*) is used in traditional medicine to treat diabetes, asthma, epilepsy, malaria, etc. Moreover, the seed or Kernel of *B. aegyptiaca* fruit is very rich in oil (46.0–54.7%) particularly unsaturated fatty acid (up to 75% of the total fatty acids) and protein (26.1–34.3%)^1,4^. Some authors successfully tested *B. aegyptiaca* oil for biodiesel production and showed that it can be an alternate diesel^5,6^.

Due to its extreme resistance to drought as well as its diverse set of natural regeneration strategies through seeds, suckers and rejuvenation, *B. aegyptiaca* has recently identified as one of the native plants for restoring of degraded Sahelian ecosystem in the so called Great Green Well (GGW) project^7^. This project aims to create a 15-km- wide (north–south) green belt of trees south of the Saharan desert across more than 11 African countries (from Senegal to Djibouti)^8,9^.

With two-third of its surface areas lying in the African great Sahara Desert, Mauritania like many other Sahelian countries was faced during the 1970s–1980s with the severe drought resulting in huge socio-economic and environmental impacts (massive rural exodus, food insecurity and desertification). The limited biodiversity that naturally characterizes arid and desert regions was significantly reduced due to that environmental condition.

Studying genetic variability is of great relevance in plant genetic resource management programs. Through information it provides, it is possible to identify genotypes of interest and use them in the establishment of effective conservation strategies.

Today, molecular markers are by far more suitable to analyze the genetic diversity than morphological and biochemical traits because they segregate as a single gene and they are not affected by the environment^10^. A vast array of molecular markers including restriction fragment length polymorphism (RFLP), randomly amplified polymorphic DNA (RAPD), amplified fragment length polymorphism (AFLP), inter simple sequence repeats (ISSR) and microsatellites or simple sequence repeats (SSR) have been used to assess genetic diversity in plant species from different parts of the world^11–15^. ISSRs are segments of DNA that are flanked at both side by shorts DNA motifs of 2–5 nucleotide long repeated multiple time called microsatellite^16^. PCR-ISSR is a quick, cost-effective and high reproducibility molecular technique based on PCR amplification of such multilocus inter-microsatellite sequences ISSRs are found to be useful in the analysis of genetic variation below the species level, mainly in studying population structure and differentiation^17^. Data on the genetic diversity of *B. aegyptiaca* germplasm worldwide are very limited. Domyati et al.^29^ assessed genetic diversity in 7 medicinal plants, including *B. aegyptiaca* using ISSR, RAPD, and AFLP. They showed that ISSR markers revealed high genetic diversity in *B. aegyptiaca* compared to other markers (RAPD, AFLP). Moreover, AFLP markers have been successfully used to assess genetic diversity amongst *B. aegyptiaca* collected from different geographical regions^18^.

In the present study, we evaluate genetic diversity among and within *B. aegyptiaca* populations collected from different bioclimatic zones in Mauritania using ISSR markers. The main objective of this study was to provide baseline molecular information for this economically and ecologically important neglected crop for a best conservation and restoration program of Sahelian ecosystem.

## Results

### Polymorphism of the ISSR markers

The 16 ISSR primers used generated 505 polymorphic bands ranged in size between 40 and 3369 bp. The primer BTH3[(AG)_8_TC] produced the lowest number of bands (21) whereas the primer BTH2[(AG)_8_T] showed the highest number (42) with an average of 31.5 fragments per primer (Table 1). The PIC values ranged from 0.13 to 0.29 with an average of 0.23. Marker Index (MI) and resolution power (Rp) averaged 7.28 (range 3.3–10.3) and 9.9 (range 4.5–14.6), respectively.

**Table 1:** ISSR primer used and their respective markers performance indexes.

Primers	Sequence 5′ → 3′	TNB	NPB	PIC	MI	RP
BTH1	(AG)8C	31	31	0.16	5.0	6.9
BTH2	(AG)8T	44	44	0.22	9.6	12.8
BTH3	(AG)8TC	21	21	0.16	3.3	4.5
BTH4	(AG)8CA	38	38	0.25	9.7	13.7
BTH5	(AG)8TA	28	28	0.26	7.3	9.9
BTH6	(AG)8CC	36	36	0.29	10.3	14.6
BTH8	(CT)8G	35	35	0.13	4.7	5.9
BTH9	(CT)8T	26	26	0.28	7.3	10.1
BTH10	(GA)8C	32	32	0.27	8.6	11.8
BTH11	(GA)8CT	36	36	0.17	6.0	7.7
BTH12	(GA)8TT	28	28	0.28	7.9	11.2
BTH13	(GT)8C	24	24	0.26	6.3	8.2
B1	(GT)6CC	31	31	0.26	8.0	10.8
B2	(GT)6GG	28	28	0.25	6.9	9.3
TH1	(GT)6CG	32	32	0.23	7.4	10.0
TH2	(GT)6TG	35	35	0.23	8.1	11.1
Average		31.5	31.5	0.23	7.3	9.9

*TNB* total number of bands, *NPB* number of polymorphic bands, *PIC* polymorphism information content, *MI* marker index, *RP* resolving power.

At the population level, the number of observed alleles (Na) ranged from 0.62 to 1.39 with an average of 1.12, while the number of effective alleles (Ne) ranged from 1.13 to 1.23 with an average of 1.2 (Table 2) and the private alleles (Pa) ranged from 2 to 14 with an average 8.29. Populations from the Saharo–Sahelian zone (Aghchorguit and Boutilimit) had the lowest (2) and the highest (14) number of private alleles, respectively. Shannon index (I) ranged between 0.13 and 0.24 with an average of 0.21. The highest percentage of polymorphic loci (%P = 68.32%) was observed in the population of Yaghref 2 and the lowest (%P = 30.1%) in the population of Tazyazet (Table 2).

**Table 2:** Genetic diversity within *B. aegyptiaca* populations from Mauritania.

Code number	Population	Na	Ne	Pa	I	%P
1	Aleg	1.3	1.23	9	0.24	64.75
2	Yaghref_1	1.36	1.23	8	0.24	67.33
3	Yaghref_2	1.39	1.21	9	0.24	68.32
4	Boutilimit	1.3	1.23	14	0.24	64.16
5	Agchorguit	1.23	1.21	2	0.22	61.19
6	Tazyazet	0.62	1.14	5	0.13	30.10
7	Chami	0.63	1.13	11	0.13	30.30
Average		1.12	1.2	8.29	0.21	55.16

*Na* observed number of alleles, *Ne* effective number of alleles, *Pa* private alleles, *I* Shannon’s information index, *%P* percentage of polymorphic loci.

### Genetic differentiation and gene flow

The coefficient of genetic differentiation (G_ST_) between populations and gene flow (Nm) were 0.21 and 1.91, respectively. Analysis of molecular variance showed that 20% of the molecular variance was between populations (Table 3).

**Table 3:** AMOVA for *B. aegyptiaca* populations from Mauritania.

Source	df	SS	MS	Est. Var.	% of variation
Among populations	6	1,209,682	201,614	11,893	20
Within populations	84	4,087,373	48,659	48,659	80
Total	90	5,297,055		60,552	100

*df* degree of freedom, *SS* sum of squares, *MS* mean of squares, *Est. Var.* estimated variation, *% of variation* percentage of variation.

### Cluster analysis

**Figure 1:**
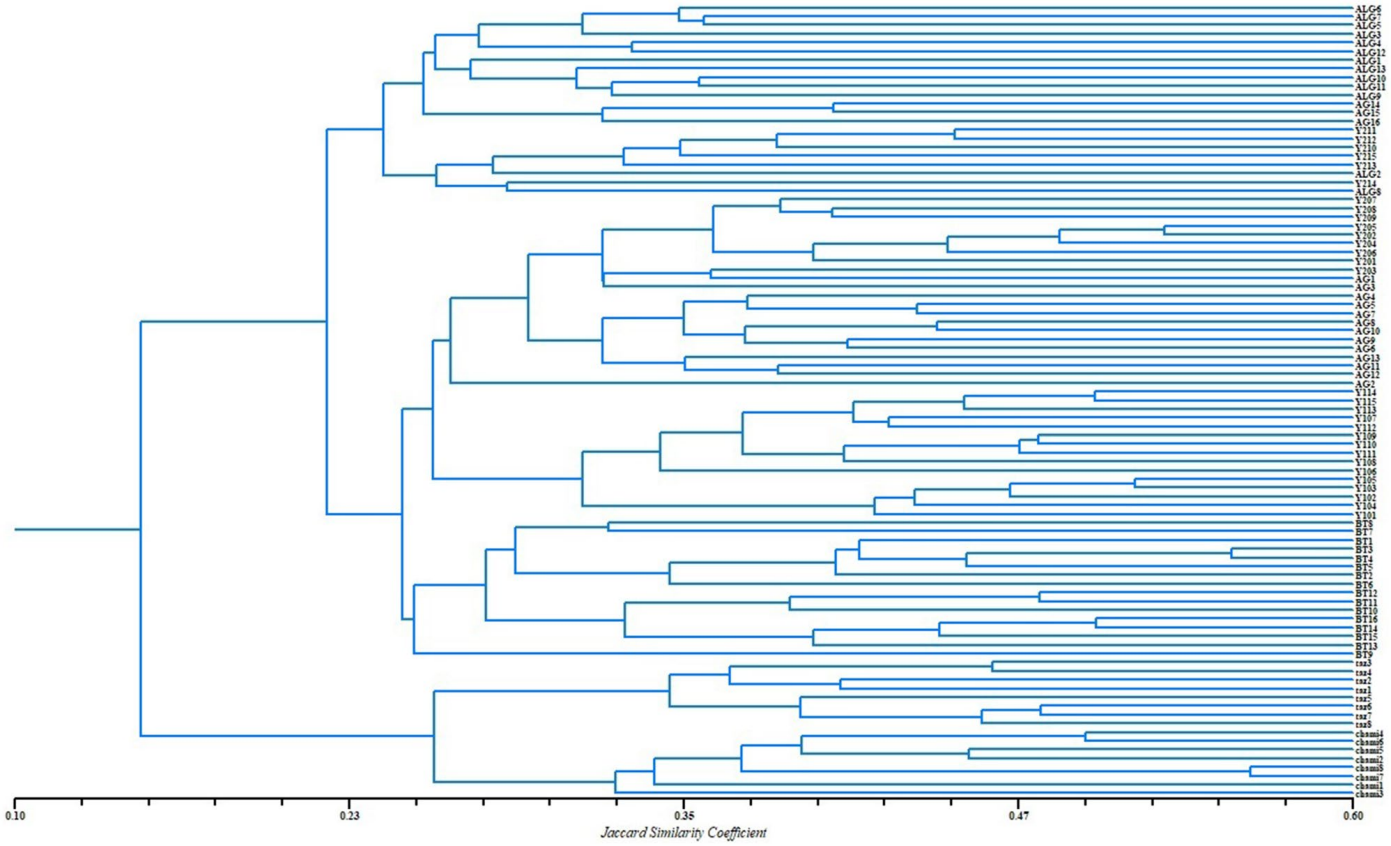
UPGMA-dendrogram based on Jaccard’s similarity coefficient, of 7 natural populations of *B. aegyptiaca,* implemented by the software NTSYS-PC version 2.02 (Exeter software, New York).

The dendrogram based on Jaccard's similarity coefficient ranging from 0.14 to 0.56 was constructed using the whole ISSR data matrix (Fig. 1). The obtained dendrogram divided the populations in to two main groups, the largest one contains five geographically distant populations (Aleg, Aghchorguit, Boutilimit, Yaghref1, Yaghref2) and the second group contains Tazyazet and Chami populations. Tazyzet and Chami both from the Saharan zone.

The dendrogram grouped the studied populations into two major clusters and the individuals were separated according to their populations. Some populations of each region were placed into the same sub-cluster and showed high similarities with each other.

### Principal coordinates analysis

**Figure 2:**
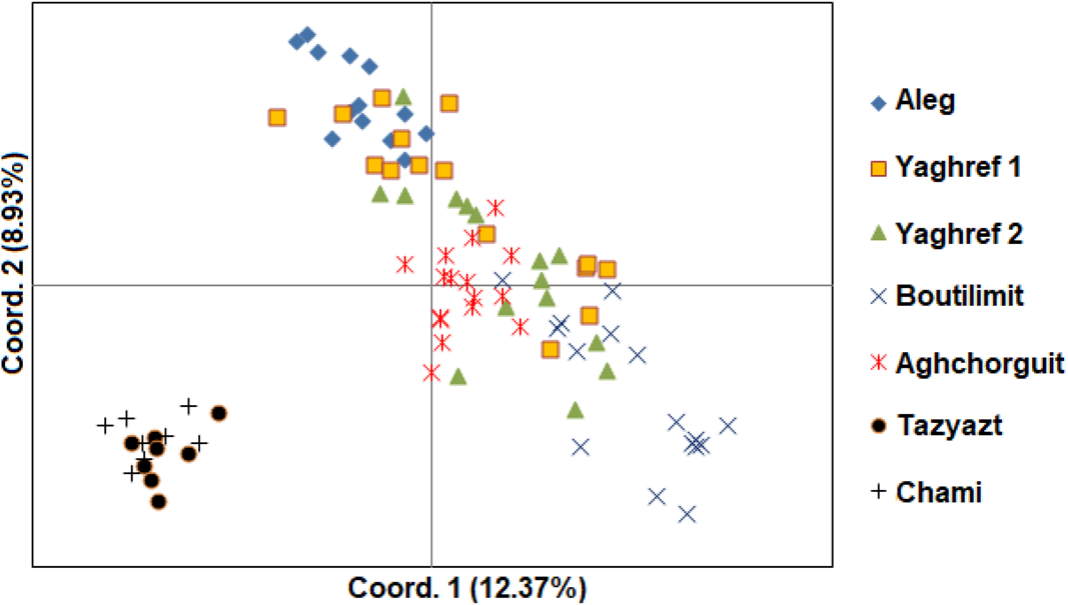
Two-dimensional representations of the first two axes of the principal coordinates analysis (PCoA) from the matrix of genetic distances of 91 samples from 7 populations.

Principal coordinates analysis (PCoA) was carried out to provide spatial representation of the genetic diversity among the desert date populations (Fig. 2). The first two principal coordinates accounted for 21.3% of the total variance (12.37% and 8.93%, respectively). Obtained picture confirmed the clustering pattern observed using UPGMA analysis and the classification of the *B. aegyptiaca* populations into two major groups. Indeed, populations of Chami and Tazyazet from the Saharan zone are genetically distant from the rest of the populations (Aleg, Agchorguit, Boutilimit, Yaghref 1 and Yaghref 2).

### Population structure analysis

**Figure 3:**
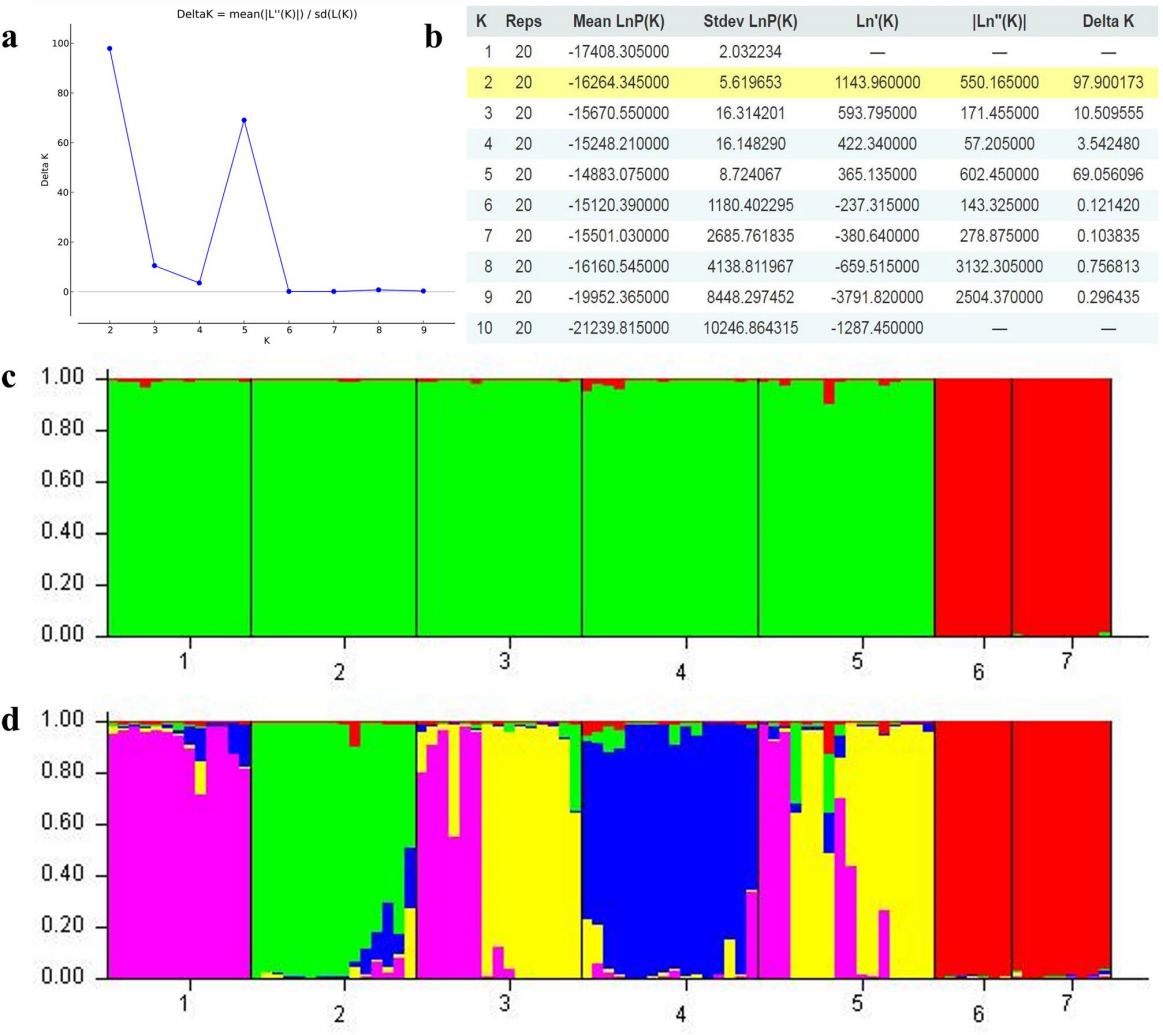
Results of Bayesian structure of the populations of *B. aegyptiaca* obtained with the structure program^37^ The STRUCTURE HARVESTER based on the approach of Evanno et al.^27^ indicates Delta K was achieved its highest peak when K = 2 followed by K = 5 (**a**). (**b**) Table output of the Evanno method results which Yellow highlight shows the maximum value in the Delta K column. From top to bottom the clusters at K = 2 (**b**) and at K = 5 (**c**). Each population is represented by a single vertical bar. The bar is divided into K colors, where K represents the number of genetic groups assumed as identified by the STRUCTURE program. Population’s numbers see Table 2.

To provide further evidence and deduce population structure, Bayesian assignment analyses were used. The maximum log-likelihood given by STRUCTURE and ∆K method was K = 2 followed by K = 5 indicating that the populations of *B. aegyptiaca* studied could be grouped into two main populations and five subpopulations (Fig. 3). The populations Tazyazet and Chami form one cluster at K = 2 and at K = 5. Whereas the populations Aleg, Agchorguit, Boutilimit, Yaghref 1 and Yaghref 2 are more similar among them and form one genetic group at K = 2. This group was further separated into 4 sub-clusters at K = 5. The above analyses (UPGMA and PCoA) indicate a similar result and show two main clusters which is consistent with the STRUCTURE results at K = 2.

## Discussion

*B. aegyptiaca* is a woody plant endemic to Mauritania as well as the Sahel where it plays an important ecological and socio-economic role. In the present study, genetic diversity among seven *B. aegyptiaca* natural populations from different bioclimatic stages in Mauritania was assessed using a set of ISSR primers. The study revealed significant number of markers (505 polymorphic bands) in the 16 tested ISSR primers compared to that reported in *B. aegyptiaca* species from Egypt (177 bands and 17 ISSR primers)^29^. Khamis et al.^18^ reported 477 polymorphic bands using AFLPs markers in *B. aegyptiaca* from different geographical origins. However, polymorphism information content (PIC) obtained in our study (0.23), is lower than that reported by Domyati et al.^29^ who found a PIC value of 0.37. Variable PICs values from other perennial dicotyledonous species such as jujub (*Z. mauritiana*) (PIC = 0.42) and cork oak (*Quercus suber *L.) (PIC = 0.28) have been reported^30,31^. Moreover, marker index (MI) for the present study populations (7.3) was very low compared to that of 53.34 found by Domyati et al.^29^ in *B. aegyptiaca* using ISSR marker. The difference in the markers in formativeness and performance between our study and that of Domyati et al.^29^ could be the results of the difference in the primers sequences used and the genetic background of the populations tested. Nevertheless, the level of polymorphism found in our study is consistent with that reported for similar long-lived perennial species such as Cork Oak^31^ and Baobab (*Adansonia digitata*)^32^.

Moreover, natural populations of *B. aegyptiaca* in the Saharan zone are subjected to several anthropogenic (the need of wood for fuel), animal (mainly dromedary grazing) and environmental (extreme aridity) pressures, that could be probably the cause of the low observed variability. It is worth noting that *B. aegyptiaca* trees in this desert region, which probably constitutes the northern limit of the species distribution in the country, are much dispersed and not abundant.

In addition, private alleles, particularly in the Saharan and Saharo-Sahelian populations, were noted. These alleles reveal information about the differentiation of species and could be involved in adaptation of the species to local environment. Also, the private alleles occur as a result of mutation^33^ and can be studied to reveal genes of adaptation. These results can be used to identify the ecotypes of this tree.

The AMOVA values also provide an insight into intra- and inter-population differentiation of *B. aegyptiaca.* Indeed, AMOVA showed that 80% of the total genetic variations was attributed to variations within populations rather than between populations (G_ST_ = 0.21). These findings are comparable to those reported by Alansi et al.^14^ in *Z. spina-christi* L. (G_ST_ = 0.17) and Medhi et al.^34^ in *Xanthoxylum* spp. (G_ST_ = 0.24). Furthermore, it was observed that 100% of the total variation in *B. aegyptiaca* populations from Egypt was attributed to difference within populations^29^. The high level of the intra-population diversity in *B. aegyptiaca* corroborate that long-lived, cross-pollinated and widespread plant species maintains high level of intra- population differentiation and low level of inter-population diversity^35^ For instance, an allopollination rate of 37% for *B. aegyptiaca* from Senegal was reported. This cross-pollination is brought about by wind and insects^36^. It is worth noting that geographically distant populations are genetically related as revealed by principal coordinates analysis, UPGMA dendrogram, based on Jaccard's similarity coefficient, and population structure analysis. This finding can be explained by the absence of geographical barriers between the regions studied and effective gene flow (Nm = 1.91). One factor that could maintain gene flow between geographically separated populations is seed dispersal through animal grazing^35^. In the case of *B. aegyptiaca*, one of the common preferred forage species of Camel dromedary, seeds can be transferred as Camel graze from one land to another during transhumance across Sahara, thus explaining why this species is common in the Sahel and Sahara.

To our knowledge, this is the first study addressing the genetic diversity of *B. aegyptiaca* from Mauritania. It demonstrates that ISSR markers offer a useful approach for characterizing genetic diversity within and among *B. aegyptiaca* populations. Further studies for analyzing genetic diversity using other ISSR primers and *B. aegyptiaca* populations may help the selection of plants of interest particularly in the context of the GGW project. Furthermore, it is useful to conduct comparative studies with different molecular and even morphological marker.

## Materials and methods

### Plant material

**Figure 4:**
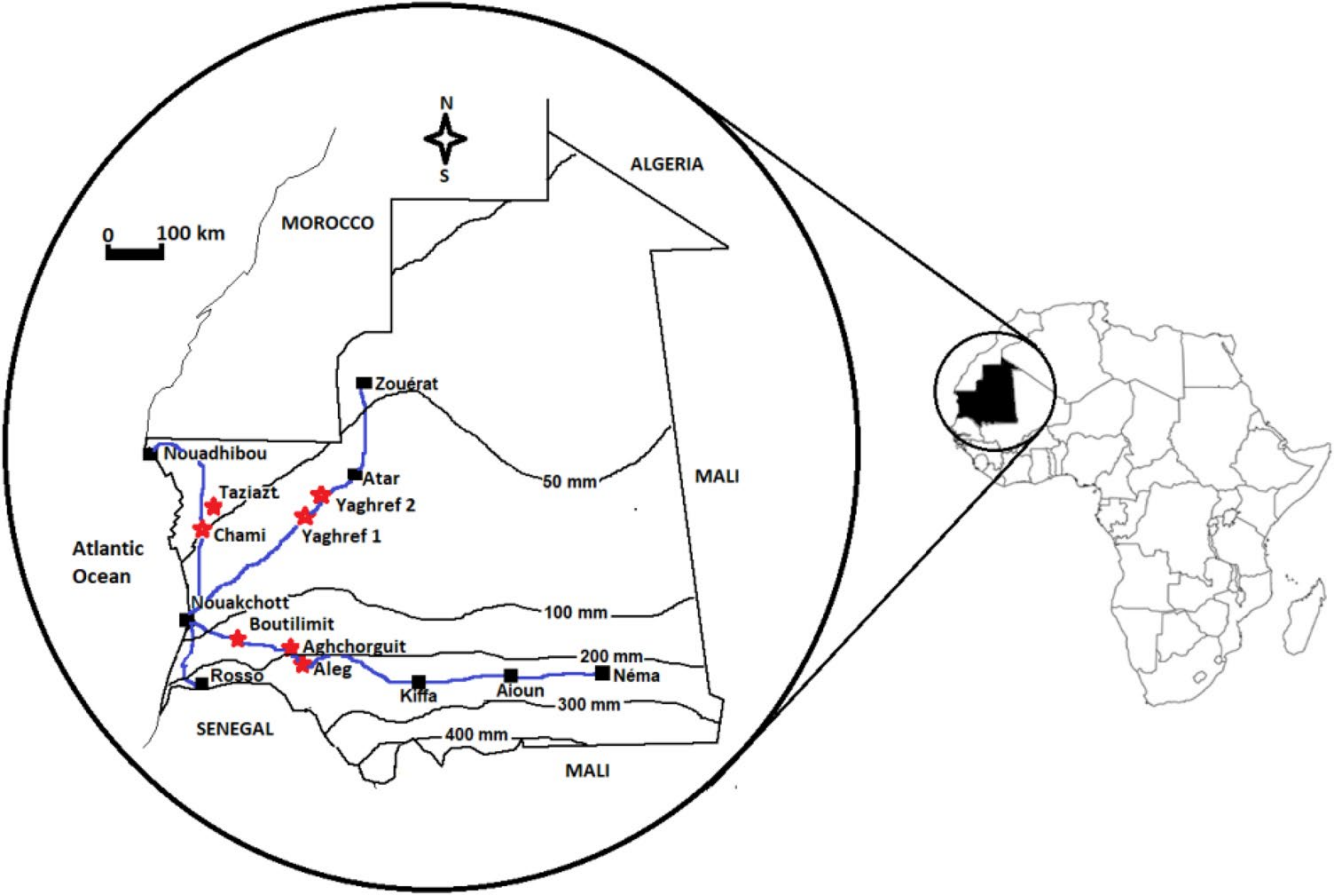
Map of Mauritania showing the study sites and rainfall patterns.

In this study, we evaluated 91 accessions belonging to 7 natural populations of *B. aegyptiaca*. These populations were collected from Aleg in the Sahelian zone (rainfall > 200 mm), Boutilimit, and Aghchorguit, in the Saharo-Sahelian zone (rainfall between 100 and 200 mm) and Yaghref1, Yaghref2, Tazyazt and Chami from the Saharan zone (rainfall < 100 mm) (Fig. 4).

The fresh leaves were collected from 10 to 15 trees per population and per study sites. Due to the asexual reproduction mode of the desert date tree through suckering^19^, a minimum distance of not less than 20 m was maintained between sampled trees for the majority of populations. The leaves were transported in a container in presence of ice to the laboratory where they were either stored at − 80 °C or lyophilized for further processing.

### DNA extraction

Genomic DNA was extracted from 20 mg of lyophilized leaves using the commercial Isolate II Plant DNA Mini Kit (Bioline, France) according to the manufacturer’s instructions. DNA integrity and purity were estimated using a spectrophotometer (NanoDrop 2000, USA) and 1% agarose gel electrophoresis.

### DNA amplification by ISSR

We tested 17 anchored ISSR primers of which 16 given reproducible bands. Table 1 summarizes the main characteristics of the ISSR primers used. PCR was carried out according to the protocol of Zietkiewicz et al.^20^ with slight modifications. Briefly, 25 µl of PCR mixture contained 4 ng/µl genomic DNA, 1U of Taq DNA Polymerase, 1 × PCR buffer, 2 mM of each dNTPs, 2.5 mM MgCl_2_ and 4 µM of each primer. DNA amplification was performed in a 96-well thermal cycler (Veriti^®^, California, USA). Cycling included a 5 min initial denaturation at 94 °C, following by 32 cycles of: 30 s at 94 °C, 45 s at annealing temperature of each primer, 2 min at 72 °C and a final elongation cycle of 7 min at 72 °C. The ISSR-PCR products were separated on 2.8% agarose gel, stained with ethidium bromide and visualized under UV. Band size was estimated by comparing the bands with 1 kb ladder (Invitrogen, USA).

### Data analysis

The binary data matrix was analyzed using GelCompar II software (version 2.5, Applied Maths, Kortrijk, Belgium). Only clear and sharp bands were considered as ISSR markers. They were then coded as 1 for the presence and 0 for the absence. From this binary presence/absence matrix, we calculated the polymorphism information content (PIC) according to the formula, described by Roldán-Ruiz et al.^21^, PICi = 2fi (1 − fi), where PICi is the polymorphic information content of marker *i*, f*i* is the frequency of the present fragments and (1 − f*i*) is the frequency of the absent fragments. Marker index (MI) was calculated according to the Powell et al.^22^ formula: MI = PIC × EMR, where EMR (effective multiple ratios) is defined as the product of the fraction of polymorphic loci (β) and the number of polymorphic loci (n). The resolving power (RP) for each primer was calculated using the formula of Prevost and Wilkinson^23^: Rp = ΣIb, where Ib is the informative fragment and calculated as follows: Ib = 1 − [2 ×|0.5 − p|], where p is the proportion of the genotypes containing the fragment.

To estimate genetic diversity, five parameters were calculated using GenAlex v6.5^23,24^ including the number of alleles (Na), the effective alleles (Ne), the private alleles (Pa), the Shannon’s information index (I) and the percentage of polymorphic loci (%P).

POPGENE version 1.32^25^ was used to compute the coefficient of gene differentiation (Gst) and the gene flow (Nm). Principal coordinate analysis (PCoA) and Molecular Analysis of Variance (AMOVA) were also calculated using the GenAlex v6.5 program.

The data were analyzed using the SIMQUAL (similarity for qualitative data) method to generate Jaccard similarity coefficients. These similarity coefficients were used to construct dendrogram using the Unweighted Pair-Group Method with Arithmetic mean (UPGMA) employing the SAHN (Sequential Agglomerative Hierarchical and Nested clustering) routine from NTSYS-PC v. 2.02 program (Applied Biostatistics, Setauket, N.Y.)^26^ The genetic structure pattern was analyzed using a Bayesian algorithm implemented in the STRUCTURE 2.3.4 software which to infer the number of genetically distinct clusters (K) without a priori group designation. Twenty independent simulations were carried out for each simulated value of K (the number of populations). The range of possible clusters was set from 1 to 10. Analysis parameters included a burn-in period of 30,000 iterations and 50,000 Markov Chain Monte Carlo (MCMC) repetitions with the selection of admixture and correlated allele frequencies models. The credible number of populations was assessed using the Evanno’s ΔK method^27^ based of maximum value of ΔK with online tool Structure Harvester^28^.

## Data Availability

All relevant data are within the paper.

## References

[1] Mohamed AM, Wolf W, Spiess WEL (2002). Physical, morphological and chemical characteristics, oil recovery and fatty acid composition of *Balanites**aegyptiaca* Del. kernels. Plant Foods Hum. Nutr..

[2] Niang K (2014). Revisiting tree species availability and usage in the Ferlo region of Senegal: a rationale for indigenous tree planting strategies in the context of the great green wall for the sahara and the Sahel initiative. J. Exp. Biol. Agric. Sci..

[3] Chothani D, Vaghasiya H (2011). A review on *Balanites aegyptiaca* Del (desert date): phytochemical constituents, traditional uses, and pharmacological activity. Pharmacogn. Rev..

[4] Khalil MI, Khalil IM (2017). Re-evaluation of fatty acids composition, physiochemical properties and thermal stability of Sudan *Balanites aegyptiaca* (Lalob) fruit oil. Agric. Biol. J. N. Am..

[5] Naik NS, Balakrishna B (2018). Assessment of toxic content in *Balanites aegyptiaca* seed cake and use of enzymatic-catalysed biodiesel in diesel engine. Int. J. Ambient Energy.

[6] Sunil Naik N, Balakrishna B (2018). Effects of EGR on performance and emissions of a diesel engine fuelled with *balanites aegyptiaca*/diesel blends. Int. J. Ambient Energy.

[7] Niang K, Ndiaye O, Diallo A, Guisse A (2014). Flore et structure de la végétation ligneuse le long de la Grande Muraille Verte au Ferlo, nord Sénégal. J. Appl. Biosci..

[8] Duponnois, R. *La Grande Muraille Verte: Capitalisation des recherches et valorisation des savoirs locaux* (IRD Éditions, 2013).

[9] Sagna MB, Niang KS, Guisse A, Goffner D (2014). *Balanites**aegyptiaca* (L.) Delile: distribution géographique et connaissances ethnobotaniques des populations locales du Ferlo (nord Sénégal). Biotechnol. Agron. Soc. Environ..

[10] Kumar P, Gupta VK, Misra AK, Modi DR, Pandey BK (2009). Potential of molecular markers in plant biotechnology. Plant Omics.

[11] Miller JC, Tanksley SD (1990). RFLP analysis of phylogenetic relationships and genetic variation in the genus Lycopersicon. Theor. Appl. Genet..

[12] Williams JG, Kubelik AR, Livak KJ, Rafalski JA, Tingey SV (1990). DNA polymorphisms amplified by arbitrary primers are useful as genetic markers. Nucleic Acids Res..

[13] Herselman L (2003). Genetic variation among Southern African cultivated peanut (*Arachis**hypogaea* L.) genotypes as revealed by AFLP analysis. Euphytica.

[14] Alansi S, Tarroum M, Al-Qurainy F, Khan S, Nadeem M (2016). Use of ISSR markers to assess the genetic diversity in wild medicinal *Ziziphus**spina-christi* (L.) Willd. collected from different regions of Saudi Arabia. Biotechnol. Biotechnol. Equip..

[15] Mouhaddab J (2017). Using microsatellite markers to map genetic diversity and population structure of an endangered Moroccan endemic tree (*Argania**spinosa* L. Skeels) and development of a core collection. Plant Gene.

[16] Ng WL, Tan SG (2015). Inter-simple sequence repeat (ISSR) markers: are we doing it right. ASM Sci. J..

[17] Wei L, Wu X-J (2012). Genetic variation and population differentiation in a medical herb *Houttuynia**cordata* in China revealed by inter-simple sequence repeats (ISSRs). Int. J. Mol. Sci..

[18] Khamis G, Schaarschmidt F, Papenbrock J (2017). Genetic diversity among populations of the xerophytic tree species *Balanites aegyptiaca* and its morpho-physiological responses to water deficiency. Afr. J. Agric. Res..

[19] Tchiagam J-BN, Ndzié J-P, Bellefontaine R, Mapongmetsem P-M (2011). Multiplication végétative de *Balanites**aegyptiaca* (L.) Del., *Diospyros**mespiliformis* Hochst. Ex. A. Rich. et *Sclerocarya**birrea* (A. Rich.) Hochst. au nord du Cameroun. Fruits.

[20] Zietkiewicz E, Rafalski A, Labuda D (1994). Genome fingerprinting by simple sequence repeat (SSR)-anchored polymerase chain reaction amplification. Genomics.

[21] Roldàn-Ruiz I, Dendauw J, Van Bockstaele E, Depicker A, De Loose M (2000). AFLP markers reveal high polymorphic rates in ryegrasses (*Lolium* spp.). Mol. Breed..

[22] Powell W (1996). The comparison of RFLP, RAPD, AFLP and SSR (microsatellite) markers for germplasm analysis. Mol. Breed..

[23] Prevost A, Wilkinson MJ (1999). A new system of comparing PCR primers applied to ISSR fingerprinting of potato cultivars. Theor. Appl. Genet..

[24] Peakall R, Smouse PE (2012). GenAlEx 6.5: genetic analysis in Excel. Population genetic software for teaching and research—an update. Bioinformatics.

[25] Yeh, F. C., Yang, R. C., Boyle, T., Ye, Z. H. & Mao, J. X. POPGENE, version 1.32: the user friendly software for population genetic analysis. *Mol. Biol. Biotechnol. Cent. Univ. Alta. Edmont. AB Can.* (1999).

[26] Rohlf FJ (1997). NTSYS-pc Version. 2.02 i Numerical Taxonomy and Multivariate Analysis System.

[27] Evanno G, Regnaut S, Goudet J (2005). Detecting the number of clusters of individuals using the software STRUCTURE: a simulation study. Mol. Ecol..

[28] Earl DA, von Holdt BM (2012). STRUCTURE HARVESTER: a website and program for visualizing STRUCTURE output and implementing the Evanno method. Conserv. Genet. Resour..

[29] Domyati FM (2011). Molecular markers associated with genetic diversity of some medicinal plants in Sinai. J. Med. Plants Res..

[30] Singh AK (2007). Assessment of genetic diversity in *Ziziphus**mauritiana* using inter-simple sequence repeat markers. J. Plant Biochem. Biotechnol..

[31] Laakili A (2018). Diversity and spatial genetic structure of natural Moroccan *Quercus susber* L. assessed by ISSR markers for conservation. Physiol. Mol. Biol. Plants.

[32] Munthali CRY, Chirwa PW, Changadeya WJ, Akinnifesi FK (2013). Genetic differentiation and diversity of *Adansonia digitata* L. (baobab) in Malawi using microsatellite markers. Agrofor. Syst..

[33] Mariette S, Le Corre V, Austerlitz F, Kremer A (2002). Sampling within the genome for measuring within-population diversity: trade-offs between markers. Mol. Ecol..

[34] Medhi K, Sarmah DK, Deka M, Bhau BS (2014). High gene flow and genetic diversity in three economically important *Zanthoxylum* spp. of Upper Brahmaputra Valley Zone of NE India using molecular markers. Meta Gene.

[35] Hamrick JL, Godt MJW, Sherman-Broyles SL (1992). Factors influencing levels of genetic diversity in woody plant species. New For..

[36] Ndoye M, Diallo I, Gassama YK (2004). Reproductive biology in *Balanites aegyptiaca* (L.) Del., a semi-arid forest tree. Afr. J. Biotechnol..

[37] Pritchard JK, Stephens M, Donnelly P (2000). Inference of population structure using multilocus genotype data. Genetics.

